# Screening Driving Transcription Factors in the Processing of Gastric Cancer

**DOI:** 10.1155/2016/8431480

**Published:** 2016-06-15

**Authors:** Guangzhong Xu, Kai Li, Nengwei Zhang, Bin Zhu, Guosheng Feng

**Affiliations:** ^1^Department of General Surgery, Beijing Shijitan Hospital, Capital Medical University, Beijing 100038, China; ^2^Department of General Surgery, Beijing Chao-Yang Hospital, Capital Medical University, Beijing 100020, China

## Abstract

*Background*. Construction of the transcriptional regulatory network can provide additional clues on the regulatory mechanisms and therapeutic applications in gastric cancer.* Methods*. Gene expression profiles of gastric cancer were downloaded from GEO database for integrated analysis. All of DEGs were analyzed by GO enrichment and KEGG pathway enrichment. Transcription factors were further identified and then a global transcriptional regulatory network was constructed.* Results*. By integrated analysis of the six eligible datasets (340 cases and 43 controls), a bunch of 2327 DEGs were identified, including 2100 upregulated and 227 downregulated DEGs. Functional enrichment analysis of DEGs showed that digestion was a significantly enriched GO term for biological process. Moreover, there were two important enriched KEGG pathways: cell cycle and homologous recombination. Furthermore, a total of 70 differentially expressed TFs were identified and the transcriptional regulatory network was constructed, which consisted of 566 TF-target interactions. The top ten TFs regulating most downstream target genes were BRCA1, ARID3A, EHF, SOX10, ZNF263, FOXL1, FEV, GATA3, FOXC1, and FOXD1. Most of them were involved in the carcinogenesis of gastric cancer.* Conclusion*. The transcriptional regulatory network can help researchers to further clarify the underlying regulatory mechanisms of gastric cancer tumorigenesis.

## 1. Introduction

As one of the most common malignant tumors, gastric cancer is the third cause of cancer-related mortality worldwide, which is mainly related to late presentation. Its incidence is affected by various genetic and environmental factors, reflecting a characteristic geographical distribution. Eastern Asia, Central and Eastern Europe, and South America are higher-risk areas, whereas Northern America and most parts of Africa are low-risk areas [1]. Therefore, it is urgent to uncover the underling regulatory mechanism of gastric cancer tumorigenesis and identify the useful targets for early diagnosis and treatment.

Many researchers have devoted themselves to study the pathogenesis of gastric cancer and look for the potential targets for diagnosis and treatment. At present, several factors, such as HER2, VEGF, FEGFR, and mammalian target of rapamycin (mTOR), have been considered as targets of therapy for gastric cancer [2]. By bioinformatics method, Jian and Chen suggested that two potentially critical transcription factors, E2F1 and STAT1, may play vital roles in progression of gastric cancer [3]. Recently, the TF-miRNA coregulatory network was constructed, which provided the first evidence to illustrate that altered gene network was associated with gastric cancer invasion [4].

To date, there are still no definitive tools for the diagnosis of gastric carcinoma, due to the fact that regulatory mechanism of gastric cancer is not clarified. The integration of multiple microarray studies may be useful to provide additional evidence for understanding the regulatory mechanism. Herein, we conducted integrated analysis of gastric cancer microarray data and identified more candidate differentially expressed genes (DEGs) between gastric cancer and normal control tissues. Moreover, the significantly enriched functions of these genes were screened and analyzed to discover the biological processes and signaling pathways associated with gastric cancer. A transcriptional regulatory network was further constructed.

## 2. Materials and Methods

### 2.1. Microarray Data

Gene Expression Omnibus (GEO) database is a public functional genomics data repository (http://www.ncbi.nlm.nih.gov/geo/) [5]. The following key words were used: (“gastric cancer” [MeSH Terms] OR gastric cancer [All Fields]) AND “Homo sapiens” [porgn] AND “gse” [Filter]. The study type was defined as “expression profiling by array.” All the expression profiles were measured using the platform of Affymetrix Human Genome U133 Plus 2.0 Array. All the cancer and normal adjacent gastric tissues were obtained by resection during surgery and immediately frozen in liquid nitrogen.

### 2.2. Identifying DEGs by Information Theoretic Analysis

Firstly, the six datasets were preprocessed by background correction and normalization. Limma package [6] is the most popular method for the analysis of DEGs, and the gastric cancer and normal samples were compared using Limma package in order to identify the DEGs between the two tissue types. *P* value was determined by R software using the two-tailed Student's *t*-test [7], and the further false discovery rate (FDR) was further calculated. The gene with FDR < 0.01 was considered to indicate a DEG.

### 2.3. Functional Annotation of DEGs

In order to assess the changes in DEGs occurring at the cellular level and the functional clustering of DEGs, the enrichment analysis tool GeneCodis3 (http://genecodis.cnb.csic.es/analysis/) was used to uncover the biological meaning for groups of genes [8], including Gene Ontology (GO) categories [9] and Kyoto Encyclopedia of Genes and Genomes (KEGG) pathway annotation [10].

### 2.4. Screening the Target Sites of Potential Transcription Factors (TFs)

DEGs between gastric cancer and normal tissues could be activated or repressed by TFs. All the TFs in human genome and the motifs of genomic binding sites were downloaded from the TRANSFAC database [11]. Moreover, the position weight matrix (PWM) was also downloaded for gene promoter scanning [12]. The target sites of potential TFs were then identified. Combined with the DEGs obtained from integrated analysis, the differentially expressed targets were screened. Finally, the transcriptional regulatory network was constructed and visualized by Cytoscape software [13].

### 2.5. Online Validation of Differentially Expressed TFs

The online tool Cancer Browser (https://genome-cancer.ucsc.edu/proj/site/hgHeatmap/) was used to verify the expression of top ten differentially expressed TFs, which regulated the most downstream target genes. We selected the dataset of TCGA stomach adenocarcinoma (STAD) gene expression by RNAseq (Illumina HiSeq), in which 421 samples were enrolled, including 384 cases and 37 normal controls. The dataset ID was TCGA_STAD_exp_HiSeq. We input the names of top ten TFs in the “Genes” item on the top of screen and then clicked the “Go” button and the heat map would appear automatically, which represented the expression level for TFs in different samples.

## 3. Results

### 3.1. Identification of DEGs in Gastric Cancer

According to the inclusion criteria, we downloaded six gene expression profiles of gastric cancer from microarray experiments. GEO IDs were GSE13911, GSE19826, GSE34942, GSE35809, GSE51105, and GSE57303. Totally, there were 340 tumor samples and 43 normal gastric tissues, respectively. The types of samples were as follows: GSE13911 (26 intestinal + 6 diffuse + 4 mixed + 2 unclassified), GSE19826 (unknown Lauren subtype), GSE34942 (39 intestinal + 11 diffuse + 6 unclassified), GSE35809 (34 intestinal + 30 diffuse + 6 unclassified), GSE51105 (49 intestinal + 35 diffuse + 10 mixed), and GSE57303 (Lauren subtype not further provided). The characteristics of eligible datasets were summarized in Table 1.

Integrated analysis of six microarray datasets led to 17481 genes. Using the FDR < 0.01 as the statistical significance threshold, a total of 2327 DEGs were identified, including 2100 upregulated DEGs and 227 downregulated DEGs. The top ten upregulated and downregulated DEGs between gastric cancer and normal tissues were listed in Table 2.

### 3.2. Functional Enrichment Analysis of DEGs

GO enrichment analysis of DEGs was performed to understand their biological functions. In our present study, the three GO categories (biological process, cellular component, and molecular function) were detected, respectively, using web-based software GeneCodis3. The results of enrichment analysis showed that the significantly enriched GO terms for biological process were multicellular organismal process (GO: 32501, FDR = 1.85*E* − 09) and digestion (GO: 7586, FDR = 6.11*E* − 09) (Table 3). Moreover, the extracellular space (GO: 5615, FDR = 4.55*E* − 10) was the significantly enriched GO term for cellular component. Notably, the significantly enriched GO term for molecular functions was extracellular matrix structural constituent (GO: 0005201, FDR = 2.82*E* − 04).

Moreover, the KEGG pathway enrichment analysis indicated that cell cycle (FDR = 4.81*E* − 33) was significantly enriched (Table 4). Furthermore, several pathways were also significantly enriched which may be closely related to transcription and translation process, including spliceosome (FDR = 5.45*E* − 34), RNA transport (FDR = 8.58*E* − 23), ribosome biogenesis in eukaryotes (FDR = 5.29*E* − 22), and homologous recombination (FDR = 3.89*E* − 08).

### 3.3. Building Up TFs-Target Genes Regulatory Network for Gastric Cancer

**Figure 1:**
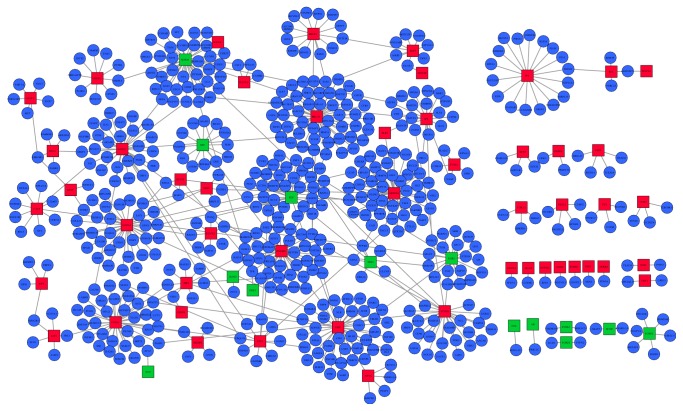
The established transcriptional regulatory network in gastric cancer. Rectangle indicates TFs, and ellipse indicates target genes. Red-color and green-color nodes represent products of upregulated and downregulated TFs, respectively. Blue nodes indicate differentially expressed target genes.

In order to display the TFs-target genes regulatory network for gastric cancer, we utilized TRANSFAC to inquire TFs and their latent target genes and then selected the differentially expressed TFs and latent target genes in gastric cancer tissues. We found a total of 70 differentially expressed TFs (54 upregulated and 16 downregulated) and 470 latent differentially expressed target genes in gastric cancer, respectively (Table 5). And, based on them, the transcriptional regulatory network was subsequently constructed. In the network, there were 63 TFs (49 upregulated and 14 downregulated) and 566 TF-target interactions in the context of gastric cancer (Figure 1). No differentially expressed target genes were found for the other seven TFs. In the network, the top ten TFs regulating most downstream target genes were BRCA1, ARID3A, EHF, SOX10, ZNF263, FOXL1, FEV, GATA3, FOXC1, and FOXD1. The three hub TFs were BRCA1 (degree = 49), ARID3A (degree = 47), and EHF (degree = 42).

### 3.4. Online Validation of Differentially Expressed TFs

**Figure 2:**
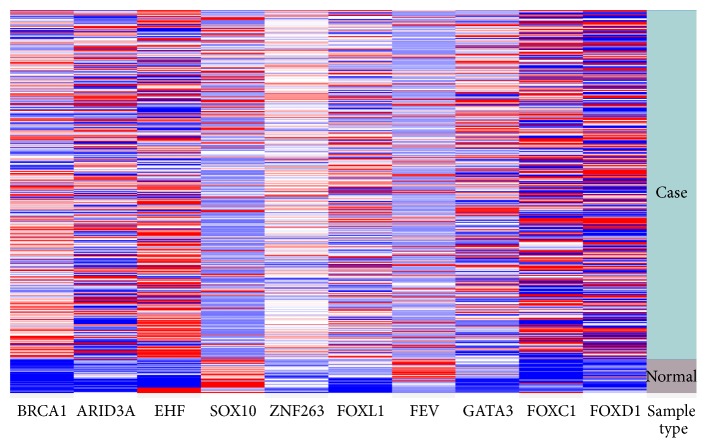
Heat map of top ten differentially expressed TFs in the dataset of TCGA stomach adenocarcinoma (STAD) gene expression by RNAseq. Sample type: green indicates the primary tumor of stomach adenocarcinoma (*n* = 384); grey indicates the normal lung tissue (*n* = 37). For each gene, red is upregulated and blue is downregulated in the corresponding sample.

The top ten differentially expressed TFs were selected for validation. The online validation revealed that expression patterns of the top ten TFs were similar to the integrated analysis. The results revealed that SOX10 and FEV were downregulated, while BRCA1, ARID3A, EHF, ZNF263, FOXL1, GATA3, FOXC1, and FOXD1 were upregulated in primary gastric adenocarcinoma compared with the normal lung tissue (Figure 2).

## 4. Discussion

Gastric cancer has few symptoms during the early stages, and most patients are usually diagnosed after the cancer has progressed to an advanced stage, which results in short survival times. Therefore, the high mortality rate underlines the need for early diagnosis and effective medical treatments for the patients [14]. The transcriptional regulatory network may be helpful to understand the underlying regulatory mechanisms and provide additional evidence for therapeutic applications.

In this study, according to integrated analysis of six microarray datasets for gastric cancer, 2327 DEGs were identified (2100 upregulated and 227 downregulated). We also observed that digestion (GO: 7586, FDR = 6.11*E* − 09) was a significantly enriched GO term for biological process. The pathway of homologous recombination was also significantly enriched, which is in accordance with the previous study where homologous recombination deficiency directly compromises the genomic stability and predisposes to cancer formation [15]. Gastric cancer is a multistep and multifactorial process, in which the dynamic balance between the cell proliferation and apoptosis of gastric mucosa was broken. Tumor suppressor gene p53 can be repressed, excessively leading to the gastric epithelial cell proliferation and the apoptosis signal cannot be started. We found that DEGs were significantly enriched in p53 signaling pathway, and various pathways related to cell proliferation were also enriched, such as cell cycle, DNA replication, pyrimidine metabolism, purine metabolism, and mismatch repair. Our results suggested that the above pathways may drive the tumorigenesis of gastric cancer.

Moreover, 70 differentially expressed TFs were identified and a transcriptional regulatory network was constructed. In the network, top ten TFs regulating most downstream target genes were BRCA1, ARID3A, EHF, SOX10, ZNF263, FOXL1, FEV, GATA3, FOXC1, and FOXD1. Most of them were involved in the progression of gastric cancer.

BRCA1 is an important tumor suppressor, which plays an essential role in maintaining genomic stability and integrity. BRCA1 was previously suggested as a good prognostic factor for gastric cancer [16]. It was reported that downregulation of BRCA1 nuclear expression was associated with advanced stage and perineural invasion in sporadic gastric cancer [17]. The loss of BRCA1 expression may serve as a predictive factor for the progression of gastric cancer [18].

ARID3A is a member of the ARID family of DNA-binding proteins. The expression of ARID3A was markedly increased in colon cancer tissue compared with matched normal colonic mucosa. A previous study suggested that strong expression of ARID3A may predict a good prognosis in patients with colorectal carcinoma, and Song et al. mentioned that whether ARID3A acts as an oncogene or tumor suppressor remains controversial [19]. In our study, we found that ARID3A was upregulated in gastric cancer compared with normal tissues. Therefore, we speculated that ARID3A may act as an oncogene in the development of gastric cancer.

Abnormalities of SOX factors have been shown to play critical roles in cancer formation and development. SOX10 was identified as a methylated gene in digestive cancers [20, 21]. It was also reported that SOX10 exhibits tumor suppressor activity by inducing tumor cell apoptosis, inhibiting invasion, and regulating cell epithelial to mesenchymal transition (EMT) in digestive cancers through suppressing Wnt/*β*-catenin signaling pathway [22]. Consistent with that, our results also indicated that SOX10 was significantly downregulated in gastric cancer, implying that the reduction of SOX10 expression could be a good predictor for gastric cancer.

The expression of FOXC1 has significance in the development, progression, and metastasis of gastric cancer, and overexpression of FOXC1 may serve as a useful marker for predicting the outcome of patients with gastric cancer [23]. Moreover, by comparative transcriptome analysis, Feng et al. found that FOXD1 was an important differentially expressed TF between metastatic gastric cancer and nonmetastatic gastric cancer [24]. Our results showed that FOXC1 and FOXD1 were upregulated in gastric cancer compared with normal tissues, which provides additional evidence for their roles of potential biomarkers.

It was reported that FOXL1 was also upregulated in pancreatic intraepithelial neoplasia [25]. Integration analysis of SNPs and gene expression profile revealed that FOXL1 regulated the most important DEGs of IRX1, SOX1, and MSX1 with risk associated SNP loci, which may serve as candidate biomarkers for diagnosis and prognosis of gastric cancer [26]. By the FOX family member such as FOXL1, hedgehog signals can induce WNT5A upregulation, which is a cancer-associated gene involved in invasion and metastasis of gastric cancer [27]. Another study reported that FOXL1 was the first mesenchymal Modifier of Min and plays a key role in gastrointestinal tumorigenesis [28]. We found that FOXL1 was upregulated in gastric cancer compared with normal tissues, indicating that FOXL1 will be a powerful driver in the progression of gastric cancer.

The expression level of GATA3 was significantly increased in patients with gastric cancer [29]. Another study reported that GATA3 plays an important role in tumor progression of gastric adenocarcinoma, and the downregulation of GATA3 is associated with unfavorable prognosis in primary gastric adenocarcinoma [30]. Specifically, Keshari et al. found that the low GATA3 expression was associated with tumors with deeper invasion, higher lymph node metastatic status, cases with distant metastases, and a later TNM stage [30]. In our study, GATA3 was upregulated in gastric cancer compared with the normal tissues, which may be partly related to the TNM stage of patients with gastric cancer. Our results suggested that GATA3 plays vital roles in different developmental stages of gastric cancer. Further functional experiments are necessary to better understand the function of GATA3 in gastric cancer.

## 5. Conclusion

Taken together, our integrated analysis discovered a bunch of DEGs in gastric cancer. Moreover, the results of function enrichment analysis revealed that some biological functions or pathways may be closely related to the development of gastric cancer, including digestion, cell cycle, and homologous recombination. The constructed transcriptional regulatory network may be helpful to further understand the underlying regulatory mechanism of gastric cancer. Ten TFs regulating most downstream target genes were obtained: BRCA1, ARID3A, EHF, SOX10, ZNF263, FOXL1, FEV, GATA3, FOXC1, and FOXD1.

## Figures and Tables

**Table 1:** Characteristics of the six microarray datasets for integrated analysis.

GEO ID	Platform	Sample (case : control)	Country	Year	Author
GSE13911	GPL570 [HG-U133_Plus_2] Affymetrix Human Genome U133 Plus 2.0 Array	38 : 31	Itay	2008	D'Errico et al. [31]
GSE19826	GPL570 [HG-U133_Plus_2] Affymetrix Human Genome U133 Plus 2.0 Array	12 : 12	China	2010	Wang et al. [32]
GSE34942	GPL570 [HG-U133_Plus_2] Affymetrix Human Genome U133 Plus 2.0 Array	56 : 0	Singapore	2014	Lei et al. [33]
GSE35809	GPL570 [HG-U133_Plus_2] Affymetrix Human Genome U133 Plus 2.0 Array	70 : 0	Singapore	2012	Lei et al. [33]
GSE51105	GPL570 [HG-U133_Plus_2] Affymetrix Human Genome U133 Plus 2.0 Array	94 : 0	Australia	2014	Busuttil et al. [34]
GSE57303	GPL570 [HG-U133_Plus_2] Affymetrix Human Genome U133 Plus 2.0 Array	70 : 0	China	2014	Qian et al. [35]

**Table 2:** Top ten upregulated and downregulated DEGs between gastric cancer and normal tissues.

Symbol	Log FC	*P* value
CST1	5.02*E* + 00	3.12*E* − 29
MMP11	3.27*E* + 00	5.35*E* − 24
COL1A1	3.27*E* + 00	1.21*E* − 30
GDF15	3.10*E* + 00	5.16*E* − 29
UBD	3.02*E* + 00	8.33*E* − 18
APOC1	3.02*E* + 00	1.03*E* − 25
SPP1	2.92*E* + 00	1.67*E* − 16
CTHRC1	2.85*E* + 00	3.95*E* − 30
COL10A1	2.84*E* + 00	1.95*E* − 19
INHBA	2.80*E* + 00	8.79*E* − 33
GKN1	−5.70*E* + 00	6.73*E* − 17
GKN2	−4.57*E* + 00	7.42*E* − 19
PGA3	−4.45*E* + 00	2.89*E* − 16
MAL	−4.19*E* + 00	1.50*E* − 33
PGA5	−3.73*E* + 00	1.24*E* − 19
ATP4B	−3.49*E* + 00	7.36*E* − 24
GIF	−3.33*E* + 00	4.22*E* − 17
ATP4A	−3.24*E* + 00	2.52*E* − 24
DPT	−3.22*E* + 00	1.67*E* − 32
C2orf40	−3.17*E* + 00	5.14*E* − 17

**Table 3:** Enriched GO terms of DEGs between gastric cancer and normal tissues.

GO ID	GO term	Number of genes	FDR
Biological process			
32501	Multicellular organismal process	58	1.85*E* − 09
3008	System process	32	1.96*E* − 09
7586	Digestion	9	6.11*E* − 09
44707	Single-multicellular organismal process	56	6.97*E* − 09
42391	Regulation of membrane potential	14	1.14*E* − 06
44057	Regulation of system process	13	1.23*E* − 06
7610	Behavior	20	1.27*E* − 06
43269	Regulation of ion transport	17	1.90*E* − 06
9719	Response to endogenous stimulus	30	2.12*E* − 06
1903522	Regulation of blood circulation	10	4.21*E* − 06
Cellular component			
5615	Extracellular space	22	4.55*E* − 10
44459	Plasma membrane part	48	1.61*E* − 09
44421	Extracellular region part	58	6.19*E* − 09
5576	Extracellular region	34	6.60*E* − 09
31226	Intrinsic component of plasma membrane	33	7.45*E* − 09
5887	Integral component of plasma membrane	31	4.49*E* − 08
31224	Intrinsic component of membrane	72	4.86*E* − 08
44425	Membrane part	85	7.79*E* − 08
1990351	Transporter complex	13	3.66*E* − 07
1902495	Transmembrane transporter complex	13	4.00*E* − 07
16021	Integral component of membrane	69	4.13*E* − 07
97458	Neuron part	31	4.20*E* − 07
34702	Ion channel complex	11	2.16*E* − 06
5886	Plasma membrane	53	2.64*E* − 06
98590	Plasma membrane region	22	3.34*E* − 05
Molecular function			
0005201	Extracellular matrix structural constituent	8	2.82*E* − 04
0043168	Anion binding	164	4.39*E* − 04

**Table 4:** Top 15 enriched KEGG pathways of DEGs between gastric cancer and normal tissues.

KEGG ID	KEGG term	Count	FDR
3040	Spliceosome	57	5.45*E* − 34
4110	Cell cycle	57	4.81*E* − 33
3013	RNA transport	51	8.58*E* − 23
3008	Ribosome biogenesis in eukaryotes	36	5.29*E* − 22
3030	DNA replication	23	8.72*E* − 18
240	Pyrimidine metabolism	32	5.19*E* − 14
230	Purine metabolism	40	1.11*E* − 12
3430	Mismatch repair	13	5.98*E* − 09
4114	Oocyte meiosis	27	2.78*E* − 08
3440	Homologous recombination	13	3.89*E* − 08
4914	Progesterone-mediated oocyte maturation	23	6.62*E* − 08
3015	mRNA surveillance pathway	22	6.82*E* − 08
4115	p53 signaling pathway	20	7.97*E* − 08
4141	Protein processing in endoplasmic reticulum	32	1.10*E* − 07
3018	RNA degradation	19	3.27*E* − 07

**Table 5:** Top ten TFs interacting with the most DEGs.

TFs	Log FC	Count	Genes
BRCA1	1.067827	49	SLC6A6, NHLRC3, TAF2, POGK, GMFB, NUSAP1, TCF20, CXCL1, FANCB, GCNT4, MPHOSPH9, TAF15, SMG1, WRAP53, HNRNPA2B1, AP1S3, ATP13A3, COPA, TMEM132A, PGM2L1, CTR9, DHX37, SAPCD2, INTS1, SLC30A7, THUMPD2, ZNF707, CCDC34, HMGN1, SLC35A2, ENPP6, CHUK, PRKCSH, ARMC10, RANBP1, LOC389906, CEP72, TIPIN, ILF3, GEMIN5, DCLRE1C, SPAG5, TRMT6, TTYH3, ZC3H11A, MIS18A, SUPV3L1, MND1, PTGES3

ARID3A	0.844259	47	MCM4, LSG1, NUP35, SPINT2, C18orf54, TASP1, REXO4, VCPIP1, AGTRAP, RFWD2, QTRTD1, PPP1R9B, CACNA2D3, ZNF207, AASDHPPT, CDC123, SLC6A4, STAMBPL1, HLF, GINS1, PIGU, TRIM37, CORO7-PAM16, ADRB2, CCNF, DDX31, TTLL5, CDH24, CAD, RPAP3, IWS1, ELK1, FBXO45, NEFL, PPP3R1, TARDBP, G2E3, AMPD1, SUPT7L, NMT1, TSLP, ORC1, FANCF, FAM213B, NUP93, TACC3, CHERP

EHF	1.300121	42	C9orf114, LRFN4, FTSJ3, LARP4, NFYA, PDRG1, ATP2A2, DPP6, ATP2C1, SNORD116-2, DCLRE1B, NME1-NME2, CENPL, ZNF146, STIL, NLK, MFAP2, DPAGT1, SNRNP200, GDF15, ATF6, UHMK1, IFI30, TRMT1, MLH3, PLBD2, PARG, ITGA2, DARS2, LY6E, KIF4A, ADPGK, USP2, TRUB1, FGFR4, BRMS1, NEIL3, ZNF598, SAFB, NCAPG2, C2orf15, MTHFD1

SOX10	−1.06478	42	YWHAB, GCA, DTYMK, TAF4, STMN1, TOP1MT, RBM12B, RAD51D, DDX10, KIFC1, CCNE2, LOC100129034, SPTAN1, DNAJC14, NUP155, SUV39H2, SNX5, SST, AJUBA, ZBTB33, CCNB1, QSOX2, NVL, NOM1, OSBPL3, ILF3, UBE2T, UBE2C, SNRPF, CBX8, PKP4, EIF3J, GCN1L1, BAZ1A, EXO1, ESRRG, ANKRD52, AGFG1, SNRNP40, TBL1XR1, SPICE1, SGOL2

ZNF263	0.435575	41	PTGES3, R3HDM1, TTYH3, RPGRIP1L, POM121, KIF2C, GABPB1, SLC7A6, ZNF526, SYMPK, KLHL12, SETDB1, PAK2, HNRNPC, POLD3, TPR, NOM1, THBS2, SULF1, SYNJ2, ATP13A2, KIF20B, CHEK1, STIP1, LRPPRC, ZMYND15, LRRC3B, MAMDC2, TNFRSF10B, SOX4, AURKAPS1, NT5C1A, TMEM199, CDK5RAP1, RAI14, SHQ1, DSCC1, ATP2A2, PTGR1, ZSCAN29, PMM2

FOXL1	0.691973	38	BCCIP, CNOT6, CCT3, CKAP2L, ZNF335, XPO5, SMARCC1, BTG2, OLFM3, PSMD12, EFCAB11, WDYHV1, PALB2, NCAPD2, TMEM5, PDRG1, FHL1, SRP72, SORCS1, TEX261, TXNDC12, ATG7, DPAGT1, HIATL1, LAMB1, UBE2O, TCOF1, NIT2, PLEKHG4, TNRC18, DUS4L, NLRC5, STAU1, TP53BP1, POLG, SSB, MMS22L, RAI14

FEV	−0.75328	37	WBP11, SH3KBP1, USP1, TIMM8A, KRT18, LTV1, ZNF485, PAK2, PODXL, ADHFE1, DIP2B, POLG2, PUS7, RCC2, DPM2, RPGRIP1L, BLOC1S2, WDR12, NCEH1, IWS1, COG2, DEPDC1, NCAM1, EPHB4, POLQ, CCT6A, MAPRE1, CENPW, SLC28A1, PIK3CB, RNF2, NSUN2, TYK2, DAZAP1, C2orf15, HN1L, SMYD5

GATA3	0.606954	35	FCHO1, ZDHHC9, CCNF, PIK3CB, TOP3A, ZNF678, EML4, WDR43, FANCM, GPN1, COL4A1, MB21D1, GORASP2, DUSP12, LGALS8, WDR3, CDC6, ZBTB41, EAF1, UFM1, HSPBAP1, PATL1, COL1A1, ARFGAP1, IKBIP, NOMO1, KAT2B, TTI1, SPG21, FAM107A, RAD51, HMMR, UHMK1, BMP1, ZC3H3

FOXC1	1.210691	32	VIT, PBK, AKAP8, ANAPC5, ILF2, NLN, RBM27, STX6, ZNF473, CHD4, MSH6, CREBZF, ZNF341, DBF4, ZNF107, PKP4, HNRNPD, CNOT6, U2SURP, CENPP, SFRP1, SUPV3L1, SFMBT1, CDKN3, NUP188, GCN1L1, NUPL1, MAMDC2, PMS1, RCCD1, UBQLN4, SMARCA5

FOXD1	0.795645	28	MTPAP, BAZ1A, CHEK1, SLC30A5, NCL, MAPRE1, AQP4, USP14, EARS2, SYNCRIP, PALB2, SLC37A3, PHF6, POLR3E, TPM3, HOXB9, CD46, CLCN5, GOLT1B, C2orf44, AAGAB, NEK2, FAM208B, MYH9, UGGT1, NOL10, PRIMA1, ZNF92

## Abbreviations

DEGs: Differentially expressed genes
GEO: Gene Expression Omnibus
GO: Gene Ontology
KEGG: Kyoto Encyclopedia of Genes and Genomes
TFs: Transcription factors.
